# Implementing lessons learned from previous bronchial biopsy trials in a new randomized controlled COPD biopsy trial with roflumilast

**DOI:** 10.1186/1471-2466-14-9

**Published:** 2014-01-31

**Authors:** Neil C Barnes, Marina Saetta, Klaus F Rabe

**Affiliations:** 1 GlaxoSmithKline, Stockley Park West, Uxbridge, Middlesex, UB11 1BT, UK and Barts and The London School of Medicine and Dentistry, London, UK; 2 Department of Cardiological, Thoracic and Vascular Sciences, Respiratory Disease Clinics,, University of Padova, Via Giustiniani 3, 35128 Padova, Italy; 3 Department of Medicine, Kiel, Germany and LungenClinic Grosshansdorf, Grosshansdorf, Germany, members of the German Center for Lung Research, University Kiel, Kiel, Germany

**Keywords:** Chronic obstructive pulmonary disease, Roflumilast, Inflammation, Exacerbation, Bronchoscopy, Bronchial biopsy, Protocol, Sputum, Histology

## Abstract

**Background:**

Chronic obstructive pulmonary disease (COPD) is a chronic inflammatory disease mediated by an array of inflammatory cells and mediators, but above all, CD8+ T-lymphocytes, macrophages and neutrophils are important players in disease pathogenesis. Roflumilast, a first-in-class, potent and selective phosphodiesterase 4 (PDE4) inhibitor, reduces the rate of exacerbations in patients with a high risk of future exacerbations and has been shown to reduce inflammatory cells and mediators in induced sputum, a surrogate of airway inflammation. However, these anti-inflammatory effects are yet to be confirmed in another robust study directly assessing inflammatory markers in bronchial sub-mucosa.

**Methods/Design:**

An international, 16-week, randomized, double-blind, placebo-controlled, parallel-group study investigating the effects of roflumilast 500 μg once-daily versus placebo on inflammatory parameters in bronchial biopsy tissue specimens, sputum and blood serum. One hundred and fifty patients with COPD and chronic bronchitis for at least 12 months will be recruited into the study and randomized in a 1:1 ratio to receive either roflumilast or placebo. The primary endpoint will be the number of CD8+ cells (cell counts per mm^2^) in bronchial biopsy tissue specimens (sub-mucosa) and the key secondary endpoint will be the number of CD68+ cells (cell counts per mm^2^), assessed by indirect immunohistochemistry.

**Discussion:**

It is hypothesized that treatment with roflumilast reduces the characteristic inflammation found in the airways of patients with moderate-to-severe COPD, compared with placebo. The design of the present study has built on the work of previous bronchial biopsy studies available in the literature. It is hoped that it will reveal the cellular mechanisms underlying the anti-inflammatory effects of roflumilast and identify potentially important biomarkers and other surrogate endpoints in patients with COPD. The design and rationale for this trial are described herein.

**Trial registration:**

Clinical trial identifier: NCT01509677 (clinicaltrials.gov)

## Background

Chronic obstructive pulmonary disease (COPD) is a major public health problem and it is projected that its burden will increase over the coming decades [1]. The Global Initiative for Chronic Obstructive Lung Disease (GOLD) document defines COPD as a “preventable and treatable disease, characterized by persistent airflow limitation that is usually progressive and associated with an enhanced chronic inflammatory response in the airways and the lung to noxious particles or gases. Exacerbations and comorbidities contribute to the overall severity in individual patients” [2].

COPD is a chronic inflammatory disease. Most notably, CD8+ T-lymphocytes, macrophages (CD68+) and neutrophils are increased in the airways and sputum of patients with chronic bronchitis and COPD [3-8]. Increased CD8+ T-lymphocyte counts have been characterized in the alveolar walls, [8] pulmonary arteries, [8] peripheral airways, [3,7] bronchial glands [6] and subepithelium [4] of patients with COPD. Moreover, neutrophil numbers are elevated in the bronchial glands and epithelium, [6] while increased macrophage infiltration has been observed in the subepithelium [4] and bronchial glands of symptomatic patients [6]. In a cross-sectional study of patients with a wide range of COPD severity, Hogg and colleagues have shown that the percentage of airways containing inflammatory cells (including neutrophils, macrophages and CD8+ cells), increases with increasing GOLD stage of COPD [3]. However, the level of inflammation underlies not only disease severity, but also exacerbation severity [9] and recovery time [10]. Papi et al. have observed that the proportion of sputum neutrophilia correlates positively with exacerbation severity, independently of bacterial or viral infections, and that sputum eosinophilia may be a good predictor of an imminent viral exacerbation [9]. There is also a significant relationship between the differences in interleukin (IL)-6 and IL-8 levels at baseline and day 7 after an exacerbation, and symptom recovery time, suggesting an important role of these inflammatory markers [10].

COPD exacerbations are also associated with increased airway and systemic inflammation [11,12]. For example, patients experiencing a severe exacerbation have augmented neutrophilic recruitment and gene expression of neutrophilic chemoattractant proteins compared to controls [11]. IL-6 and IL-8 levels are elevated in the sputum of patients experiencing an exacerbation and even in frequent exacerbators who are stable, [12] while CD8+ T-lymphocytes have been found to be increased at the onset of COPD exacerbations [13,14]. Most recently, it has been shown that CD8+ T-lymphocytes actually move from the circulation to the lung following experimental RV infection in COPD patients [15].

The use of bronchial biopsies has contributed significantly to our knowledge of COPD, helping to reveal the anti-inflammatory properties of COPD therapies, and the key role of CD8+ T-lymphocytes in COPD pathology [4,16-18]. One study demonstrated that a salmeterol/fluticasone propionate combination reduces CD8+, CD45+ and CD4+ cell numbers, as well as cells expressing genes for tumor necrosis factor-α (TNFα) [17]. Bourbeau et al. have confirmed that the same combination has anti-inflammatory effects which are not observed with use of the inhaled corticosteroid alone [16].

Roflumilast is a first-in-class, potent and selective phosphodiesterase 4 (PDE4) inhibitor, which targets the underlying chronic inflammation in COPD. As the first approved selective PDE4 inhibitor, roflumilast reduces the rate of exacerbations in patients with a high risk of future exacerbations (GOLD patient groups C and D) and symptoms of chronic cough and sputum (chronic bronchitis) [2,19,20]. A placebo-controlled clinical study has shown that roflumilast reduces absolute neutrophil and eosinophil counts in induced sputum [21]. However, evidence for its anti-inflammatory effects is limited and warrants further investigation in another study, in patients with COPD.

Accordingly, an ongoing clinical study has been designed with the aim of increasing our understanding of the anti-inflammatory effects of roflumilast. The study will analyze inflammatory markers in bronchial biopsies, induced sputum and blood serum and will offer the opportunity to identify potentially important biomarkers and surrogate endpoints in patients with COPD. The present paper brings the protocol of this clinical study to the attention of the medical community and discusses the rationale behind the study design.

## Methods

### Study design

**Figure 1:**
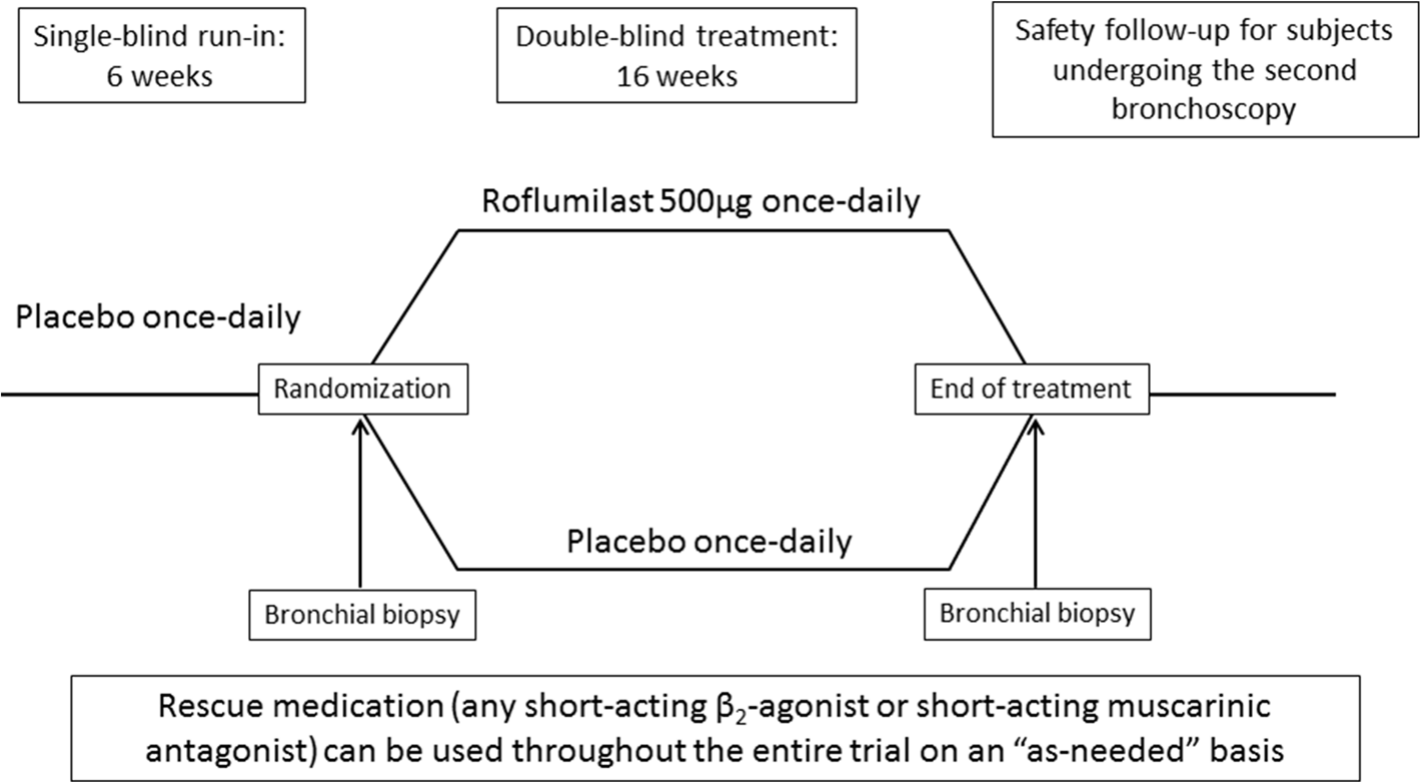
Schematic design of the clinical study.

An international, 16-week, randomized, double-blind, placebo-controlled, parallel group study (Figure 1) investigating the effect of roflumilast 500 μg once daily versus placebo on inflammation parameters in bronchial biopsy tissue specimens, sputum and blood serum (clinical trial identifier: NCT01509677). Patients will be equally randomized to either roflumilast treatment or placebo in a 1:1 ratio by means of a computerized central randomization system IVRS/IWRS. The system will assign one or two appropriate trial treatment kit(s) from the stock available at the site for each patient. The primary endpoint of the study will be the number of CD8+ cells (cell counts per mm^2^) in bronchial biopsy tissue specimens (sub-mucosa) evaluated from randomization to the end of the intervention period. The key secondary endpoint will be the number of CD68+ cells (cell counts per mm^2^), assessed over the same timeframe, but a host of other secondary outcomes will also be assessed (Table 1). The study will be conducted at 11 European sites specializing in lung diseases.

**Table 1:** Secondary endpoints evaluated from baseline or randomization to the end of the double-blind intervention period

**Biopsy material**	**Induced sputum**	**Blood serum**	**Pulmonary function changes**
Sub-mucosa cell counts (cells/mm^2^): CD68+, Neutrophils, CD4+, CD45+	Total and differential cell counts in induced sputum, absolute (cells/mL) and percentage (%): Neutrophils, Macrophages, Eosinophils, Lymphocytes	Concentration of inflammatory biomarkers: Inflammatory mediators* (Human InflammationMAP® v 1.0; Myriad RBM)	Change from randomization to the end of the intervention period:, FEV_1_FVC FEV_1_/FVC ratio
Bronchial epithelium cell counts (cells/mm^2^): CD8+ CD68+	Concentration of inflammatory biomarkers: Inflammatory mediators* (Human InflammationMAP® v 1.0; Myriad RBM)		

FEV_1_: forced expiratory volume in one second; FVC: forced vital capacity.

*Alpha-1 antitrypsin, alpha-2 macroglobulin, beta-2 microglobulin, brain-derived neurotrophic factor, complement 3, C-reactive protein, eotaxin-1, factor VII, ferritin, fibrinogen, granulocyte-macrophage colony-stimulating factor, haptoglobin, intercellular adhesion molecule-1, interferon gamma, interleukin (IL)-1 alpha, IL-1 beta, IL-1 receptor antagonist, IL-2, IL-3, IL-4, IL-5, IL-6, IL-7, IL-8, IL-10, IL-12 p40, IL-12 p70, IL-15, IL-17, IL-23, macrophage inflammatory protein-1 alpha, macrophage inflammatory protein-1 beta, matrix metalloproteinase (MMP) type 2, MMP type 3, MMP type 9, monocyte chemotactic protein-1, RANTES, stem cell factor, tissue inhibitor of metalloproteinases 1, tumor necrosis factor (TNF)-alpha, TNF-beta, TNF-receptor 2, vascular cell adhesion molecule-1, vascular endothelial growth factor, von Willebrand factor, vitamin D-binding protein.

### Ethical considerations

This trial will be conducted in accordance with the Declaration of Helsinki, Good Clinical Practice (GCP) guidelines and any additional local regulations. Ethical approval has been gained from the NRES Committee East of England - Cambridge South, UK; Regionala etikprövningsnämnden i Lund, Sweden; Komisja Bioetyczna UJ, Poland; Ethik-Kommission der Ärztekammer Schleswig-Holstein (Ethik-Kommission I), Germany as well as the UK, Swedish, Polish and German regulatory agencies.

### Patient population

Patients with a history of COPD for at least 12 months, associated with chronic productive cough for at least three months, in each of the two years prior to baseline visit will be recruited into the study. Patients with moderate-to-severe COPD (stages II and III) will be enrolled (according to GOLD 2009). Patients with a history of a recent exacerbation (within six months prior to baseline) will be excluded as will patients who have had a respiratory tract infection, which has not resolved at least four weeks before baseline. Standard bronchodilator therapy use will be permitted throughout the study. However, concomitant medications, including glucocorticoids (inhaled corticosteroids [ICSs]), oral steroids), long-acting β_2_-agonist (LABA)/ICS combinations, theophylline, lipoxygenase inhibitors, anti-platelet therapy and leukotriene antagonists will not be permitted throughout the trial and will be withdrawn at the start of the run-in period. Bronchodilators starting at least 6 weeks prior to run-in will be allowed, although these treatments must remain stable throughout the study. Other drugs for the treatment of concurrent disease will be permitted, but their doses must also be kept constant throughout study (including the run-in period). In addition to meeting the admission criteria (Table 2), patients must also satisfy the following conditions in order to be eligible for randomization into the double-blind treatment period:

• No COPD exacerbation between baseline and randomization (as defined by treatment and/or hospitalization)

• Tablet compliance ≥ 80% and ≤ 125%

**Table 2:** Main inclusion and exclusion criteria

**Main inclusion criteria**	**Main exclusion criteria**
Written informed consent obtained according to local regulations	Clinical instability (exacerbation 6 months prior to baseline as indicated by treatment)
History of COPD for at least 12 months prior to baseline, with chronic productive cough for at least three months in each of the two years prior to baseline	Upper/lower respiratory tract infection not resolved 4 weeks prior to baseline
Outpatients 40–80 years of age	Diagnosis of asthma and/or other relevant lung disease or previous episodes of pneumothorax
Post-bronchodilator 30% ≤ FEV_1_ ≤ 80% predicted	Known alpha-1 antitrypsin deficiency
Post-bronchodilator FEV_1_/FVC ratio ≤ 70%	History of intubation for COPD or a respiratory failure of any cause in the past year
Current or former smokers with smoking history ≥ 20 pack years	Formal contraindications to sputum collection
	Suspicion or diagnosis of a bleeding disorder irrespective of its pathophysiological mechanism

Information regarding patients’ COPD severity (stage II versus stage III), smoking status, concomitant LABA use and previous inhaled corticosteroid usage will be recorded for the purpose of sub-group analyses and stratification of the most important confounding variables. The number of enrolled patients will be capped at 150 in total.

Roflumilast and placebo tablets will be of identical appearance, shape and colour and will have identical labelling and packaging.

### Sample size

The sample size of 150 has been calculated from the available information on the primary endpoint and has been kept as small as possible. Previous trials have shown that drop-out rates in bronchial biopsy studies may be as high as 30% from enrolled patients; [16,17,22,23] therefore, if a conservative estimate of 30% is applied, the present study may have 105 patients who complete the trial. Drop-outs are difficult to handle in bronchial biopsy studies. If drop-outs are excluded this could unfairly bias the trial, however, if drop-outs continue in the study after having treatment with antibiotics and systemic corticosteroids, this could also bias the results. It is therefore important to choose a group of patients who are unlikely to exacerbate or drop out during the course of the trial, which is the case in our study design. A universally recognized level of clinical relevance regarding the primary endpoint (sub-mucosal CD8+ cells) has not yet been agreed on within the scientific community, but a 30% improvement using roflumilast treatment over placebo may be of clinical relevance. We have calculated that with a dispersion of 25, tissue area of 0.3 mm^2^ and further assumptions (1:1 randomization, two-sided α = 0.05, power = 0.90, event rate on roflumilast = 200 cells/mm^2^, event rate on placebo = 285 cells/mm^2^), the trial would have a high power (approximately 90%) to detect treatment differences. However, the study will not be statistically powered to investigate any outcomes with regards to the effectiveness of COPD treatment.

### Technical aspects

#### ***Bronchoscopy***

Bronchoscopies will be performed in line with the American Thoracic Society (ATS) guidelines, [24] Endobronchial Biopsy Workshop, [25] modified protocol of O’Shaughnessy et al. [4] and according to local clinical standards of care. Endobronchial biopsies will be taken from each lobar and sub-segmental carina using Olympus EndoJaw single-patient use biopsy cut forceps. In order to take into account both inter and intra-patient variability, 2–3 biopsies will be taken from the lobar bronchus and 2–3 from the sub-segmental airways, at each bronchoscopy session (randomization and end of treatment period). The left and right lobes will be alternated between subjects, but all biopsies will be harvested from the same lung during any given session. The second set of biopsies (after the treatment period) will be taken from the same airway level, but from a different specific site.

In selected patients, three protected brush specimens will be collected during the bronchoscopy procedures. If performed, these specimens will be collected prior to the bronchial biopsy procedure at the same part of the lower lobe bronchus. The specimens will be used to evaluate longitudinal changes in COPD airway microbiota in placebo-treated patients and to define the effect of roflumilast therapy on the airway microbiome.

#### ***Biopsy sample processing, cell quantification and biopsy quality***

Biopsies will be gently extracted and sent to the site’s laboratory for further processing (fixation and paraffin wax embedding). Immunostaining and quantification of inflammatory cells will be performed according to standard procedures [4,23,25,26]. Briefly, inflammatory cells will be identified using indirect immunohistochemistry (using the peroxidase-antiperoxidase complex-PAP- and diaminobenzidine as substrate or the alkaline phosphatase-anti-alkaline phosphatase complex; APAAP and Fast Red). For each antibody, the total number of positively stained cells will be counted to a depth of 100 μm below the epithelial basement membrane using a computerized image analysis.

To ensure adequate quality and consistency between investigators at different sites, centralized training, covering all aspects of material collection, handling and processing, will be provided. The quality of biopsy material will also be validated for each site, by requesting that sites provide pseudonymized samples from the first three enrolled patients. In order to be considered a good quality sample, the biopsied tissue area must be ≥0.1 mm^2^, contain ≥1 mm of basement membrane and be ≥100 μm deep. Only after the biopsy samples are considered to be of sufficiently good quality will the site be allowed to recruit further patients into the trial.

#### ***Inflammatory biomarkers in induced sputum***

Sputum will be induced, collected and initially processed at investigational sites. The quality of sputum samples will also be estimated by investigators on a scale from one to six. Total and differential cell counts of neutrophils, macrophages, eosinophils and lymphocytes will be performed and inflammatory biomarkers will be analyzed using the 46-biomarker Multi-Analyte Profiling (MAP) technology (Human InflammationMAP® v 1.0; Myriad RBM). This tool contains quantitative, multiplexed immunoassays for 46 biomarkers, but the ones of primary interest with regards to this study and roflumilast will be: alpha-2 macroglobulin, interleukin-8 (IL-8), monocyte chemotactic protein-1 (MCP-1), matrix metalloproteinase type 9 (MMP-9), tissue inhibitor of metalloproteinase (TIMP) and vascular endothelial growth factor (VEGF). The remaining 40 biomarkers will only be analyzed exploratively (Table 1). To ensure adequate quality and consistency of samples, centralized hands-on training will be provided and samples will be assessed on an ongoing basis.

#### ***Inflammatory biomarkers in blood serum***

Blood withdrawal for the measurement of inflammatory biomarkers will be performed at approximately the same time each day (±2 hours), but no later than 10:00 am at each respective visit. Inflammatory biomarkers will be quantified using MAP technology (Human InflammationMAP® v 1.0; Myriad RBM).

Blood serum, sputum and biopsy samples will be collected and appropriately stored for up to three years after the end of the study to allow for future analyses of biomarkers of scientific interest.

#### ***Pulmonary function tests***

Spirometry will be performed according to the recommendation of the ATS – European Respiratory Society (ATS/ERS) consensus guidelines on pulmonary function testing [27]. Sites will use their own devices, performing maintenance and calibration of instruments according to usual standards of practice. FEV_1_ (absolute and percentage predicted values), forced vital capacity (FVC) (absolute values) and the ratio of FEV_1_/FVC will also be recorded.

#### ***Safety***

Following bronchoscopy with bronchial biopsy, patients will be closely monitored for at least two hours and will only be discharged when the effects of sedation and local anaesthesia disappear as judged by the investigator. All patients will be provided with a 24-hour emergency contact number and a safety follow-up visit will be performed within two weeks after each bronchoscopy session.

#### ***Statistical and analytical plans***

The intention-to-treat (ITT) analysis will be based on the full analysis set (FAS). It will be the primary analysis for this study and will be performed for all primary and secondary endpoints. The primary endpoint relates to pulmonary inflammation expressed as CD8+ cell counts per mm^2^ in sub-mucosal bronchial biopsy tissue specimens measured before and after the double-blind treatment period. The key secondary endpoint relates to CD68+ cell counts per mm^2^ in sub-mucosal bronchial biopsy tissue specimens. Roflumilast/placebo comparisons for these endpoints will be performed via a multiple test procedure such that the family-wise error rate of 5% is controlled in the strong sense. The two null hypotheses are: equal CD8+ counts/mm^2^ and equal CD68+ counts/mm^2^ for roflumilast and placebo. These null hypotheses will be ordered, so that the CD8+ comparison comes first and the CD68+ comparison comes second. If the comparison with respect to CD8+ is significant at the nominal level α = 5%, the corresponding null hypothesis will be rejected and the CD68+ comparison will be performed in a confirmatory way (otherwise confirmatory testing stops). This will again be done at nominal level α = 5%. If significant (after a significant result in the first comparison), the corresponding null hypothesis will be rejected. If the first comparison is not significant at 5% level, then neither null hypothesis must be rejected. The component tests of the multiple test on CD8+ and CD68+ will be based on Poisson regression models with CD8+ (CD68+) at end of treatment period as a dependent variable and treatment and baseline values of the respective dependent variable as covariates. A dispersion parameter and an offset (equivalent to the bronchoscopy sampling area) will be taken into account.

Analyses of the other secondary endpoints will be descriptive on treatment and visit, by ANCOVA on absolute change from baseline to last available measurement during double-blind treatment for continuous variables, or Poisson regression for count data. In addition, analyses will be performed in subgroups stratified by COPD stage, smoking status, concomitant LABA and former ICS use.

## Discussion

This clinical study hypothesizes that roflumilast reduces the characteristic inflammation of COPD in patients with moderate-to-severe COPD. A specific pattern of inflammation has been characterized in the airways and lung parenchyma of COPD patients, predominantly consisting of increased numbers of CD8+ T-lymphocytes, CD68+ cells and neutrophils [3-8]. Previous research has shown that inflammatory mediators, such as IL-6 and IL-8, also play an important role in COPD [10]. Roflumilast reduces exacerbations in patients with moderate-to-severe COPD and chronic bronchitis, [19] but there are limited clinical data on its anti-inflammatory effects in the lungs as well as systemically [21]. The present study was designed to implement lessons learned from previous bronchial biopsy studies. It will hope to reveal the precise anti-inflammatory properties of roflumilast responsible for its therapeutic effect in the lungs (biopsy and sputum) and identify potentially important biomarkers and surrogate endpoints in patients with COPD. Moreover, the results from this study may also provide a better understanding of the pathophysiology of COPD and serve as a foundation for future research.

### Rationale behind the study design

This will be the first study to associate roflumilast’s effects in sputum, the lungs and systemic circulation with observed anti-inflammatory changes. Previous studies have pinpointed CD8+ T-lymphocytes and macrophages (CD68+) as cell populations characteristic in COPD inflammation [3-8]. Based on these data, CD8+ and CD68+ cell counts have been selected as the respective key primary and secondary endpoints for this study. The measurement of cell counts per mm^2^ was selected on the basis that the area profile count is a commonly used method for the quantification of inflammatory cells in bronchial biopsies [16,17,23,28]. Induced sputum offers another opportunity to obtain samples containing potentially valuable information on disease characteristics, relatively easily; while the collection of blood serum for biomarker analyses is based on the hypothesis that systemic inflammation and oxidative stress contribute to the pathogenesis of COPD. It may be argued that the endpoints used in this study are neglecting the most important site of inflammation in COPD – the small airways. However, until safe and reliable methods, which can detect small airway inflammation accurately are developed, the analysis of bronchial biopsies will remain a valuable technique for sampling COPD inflammation.

Roflumilast is effective after only four weeks of treatment, [21] and longer studies have shown similarly consistent clinical improvements [19]. Based on these data and other studies that have revealed anti-inflammatory treatment effects over 3 months, [16,17] a treatment period of 16 weeks has been selected as an optimal length of time to collect information on the anti-inflammatory effects of roflumilast, while allowing sufficient time for patients to fully recover between interventions. A 6-week single-blind run-in period has been incorporated into the study schedule to assess patients’ compliance and for reasons of standardization.

Steps have been taken to reduce factors that contribute to intra- and inter-patient variation when obtaining samples. Work by Gamble et al. has shown that endobronchial biopsies from more than one airway generation should be examined in order to maximize statistical power [29]; in this study, 2–3 endobronchial biopsies will be taken from each lobar and sub-segmental carina. At the same time, consistency between investigators and sites will be ensured by way of centralized training. Sites will also be requested to provide biopsy and sputum samples for quality evaluation before they are allowed to enrol further patients.

Selecting the most appropriate patient population for bronchial biopsy studies is an important factor. Ethically, it is not appropriate to subject patients in the indicated roflumilast patient population to the procedures required in this study, since it may put them at increased risk. Furthermore, if patients experience exacerbations during the study and require treatment with steroids or antibiotics, it would confound the analysis of the study and make it difficult to ascertain the effects of roflumilast on inflammation. With this in mind, patients with moderate-to-severe COPD (stages II and III) according to GOLD 2009 were chosen for this study. These patients were considered to be stable enough to be subject to bronchoscopy with bronchial biopsy and sputum collection. Although they are not exactly the indicated roflumilast population, it is important to note that roflumilast improves lung function in patients with stable disease too, regardless of exacerbation history [30,31]. Therefore, the study design provides us with a valuable opportunity to obtain information on the effects of roflumilast in patients with more stable disease, who have not previously been investigated [3]. All patients will continue to receive standard bronchodilator therapy throughout the trial, thereby ethically justifying the placebo arm of the study. Moreover, allowing the use of bronchodilator therapy throughout the trial will also show the effects of roflumilast on top of standard therapy.

The safety of enrolled patients is another important consideration, especially in an invasive study of this kind. Hattotuwa et al. have shown that fibre optic bronchoscopy with bronchial biopsy are procedures that can be performed in research patients with COPD (even with severe disease), with a low incidence of adverse events [32]. Nevertheless, there are risks associated with bronchoscopy such as bleeding, discomfort and coughing, although serious adverse events such as lung leak and pneumothorax are rare [33]. A number of approaches will help to minimize these safety pitfalls in this study. Firstly, bronchoscopies with bronchial biopsies will only be conducted at experienced investigational sites, which have demonstrated that they are able to perform these techniques in a standardized and safe manner, in previous clinical studies. Secondly, standardized procedures will be followed by each site. Thirdly, dedicated safety follow-up visits have been included in the study schedule and fourthly, sputum collection will alternate with bronchoscopy visits to limit the number of interventions performed on any given day. Finally, although class-specific adverse events have been reported in previous clinical studies with roflumilast, the treatment has at the same time proved to be effective in COPD patients with moderate-to-very severe airflow limitation [19,34]. Nevertheless, drug-specific adverse events will be closely monitored and recorded throughout the study.

## Conclusions

This bronchial biopsy trial will increase our understanding of the anti-inflammatory effects of roflumilast in COPD. Roflumilast reduces COPD exacerbations, but its actions in the lungs, particularly its anti-inflammatory activities, are not well understood. A better comprehension of the effects of roflumilast on inflammatory cells and mediators may help in identifying patients who would benefit most from treatment. It would also improve our understanding of which measurable parameters (e.g. cell counts, activities and mediators) might serve as surrogate predictors for the clinical efficacy of roflumilast.

## Abbreviations

APAAP: Alkaline phosphatase-anti-phosphatase complex; ATS: American Thoracic Society; COPD: Chronic obstructive pulmonary disease; ERS: European Respiratory Society; FAS: Full analysis set; FEV1: Forced expiratory volume in one second; FVC: Forced vital capacity; GCP: Good clinical practice; GOLD: Global initiative for chronic obstructive lung disease; ICS: Inhaled corticosteroid; ITT: Intention-to-treat; LABA: Long-acting β_2_-agonist; MAP: Multi-analyte profiling; MCP-1: Monocyte chemotactic protein-1; MMP-9: Matrix metalloproteinase-9; PDE4: Phosphodiesterase-4; TFNα: Tumor necrosis factor-alpha; TIMP: Tissue inhibitor of metalloproteinase; VEGF: Vascular endothelial growth factor.

## Competing interests

The study is being funded by Takeda Pharmaceuticals International GmbH.

N.B. has received lecture and consultancy fees from GlaxoSmithKline, Boehringer Ingelheim, Merck Sharp and Dohme, Nycomed/Takeda, Forest Pharmaceuticals, Almirall and Novartis. He has received research funding from GlaxoSmithKline, AstraZeneca, Almirall and Novartis. At the time of manuscript preparation, N.B. was a Professor of Respiratory Medicine at Barts and The London School of Medicine and Dentistry, UK. He has since moved to GlaxoSmithKline.

M.S. has received lecture fees, consulting fees, and a grant for research from Takeda; has received lecture fees and a grant for research from Chiesi Farmaceutici; and has received lecture fees from GlaxoSmithKline and AstraZeneca. M. Saetta does not have any other competing interests.

K.F.R. has provided legal consultation services or expert witness testimony to AstraZeneca, Chiesi Pharmaceutical, Novartis, MSD and GlaxoSmithKline. He has also received research funding from Altana Pharma, Novartis, AstraZeneca, MSD and Nycomed/Takeda.

## Authors’ contributions

All authors are investigators in the study and participated in its design and coordination and helped to draft the manuscript. All authors read and approved the final manuscript.

## Pre-publication history

The pre-publication history for this paper can be accessed here:

http://www.biomedcentral.com/1471-2466/14/9/prepub
